# Pancreatic Laceration in a Pediatric Patient: An Unexpected Diagnosis

**DOI:** 10.1155/2017/2681835

**Published:** 2017-11-02

**Authors:** Michelle J. Hong, Lauren M. Porter, Debra D. Esernio-Jenssen, Andrew C. Miller, Marna Rayl Greenberg

**Affiliations:** ^1^Department of Emergency Medicine, Lehigh Valley Hospital and Health Network, USF MCOM, CC & I-78, Allentown, PA 18103, USA; ^2^Department of Pediatrics, Lehigh Valley Hospital and Health Network, USF MCOM, CC & I-78, Allentown, PA 18103, USA

## Abstract

Pediatric pancreatic injuries are rare. We present an atypical case that occurred in a 4-year-old male. The child presented with a twenty-four-hour history of vomiting that had progressed to right lower quadrant abdominal pain on examination in the emergency department. The initial differential was gastroenteritis versus appendicitis. An abnormality on the ultrasonography and an elevated lipase level eventually led to an MRI showing a complete transection through the posterior margin of the pancreas. The patient was admitted to pediatric surgery and underwent a successful distal pancreatectomy with preservation of the spleen. On further inquiry specific to trauma, the child disclosed that his older brother had punched him in his abdomen the night before. The child's parents were separated due to intimate partner violence, and this older sibling recently had been very stressed. The sibling was referred for mental health evaluation and counseling, and the case reported to the county children and youth investigative services system. A low threshold for considering trauma and child abuse in the pediatric population is recommended when significant intra-abdominal injury is diagnosed.

## 1. Case Report

A 4-year-old male presented to the emergency department complaining of a 1-day onset of nausea and vomiting that had progressed to periumbilical abdominal pain radiating to the right lower quadrant. Physical examination revealed a blood pressure of 117/79 mm Hg, pulse 118 beat/min, respiratory rate 22 breaths/min, oxygen saturation 100%, and temperature 99.3°F. He appeared well developed and well nourished. He was alert and presented no neurologic deficits. He produced mucous vomit during examination. He exhibited tenderness upon palpation over the periumbilical and right lower quadrant regions of the abdomen. There was mild guarding with no rebound. Bowel sounds were normal, and he exhibited no distention and no mass.

**Figure 1:**
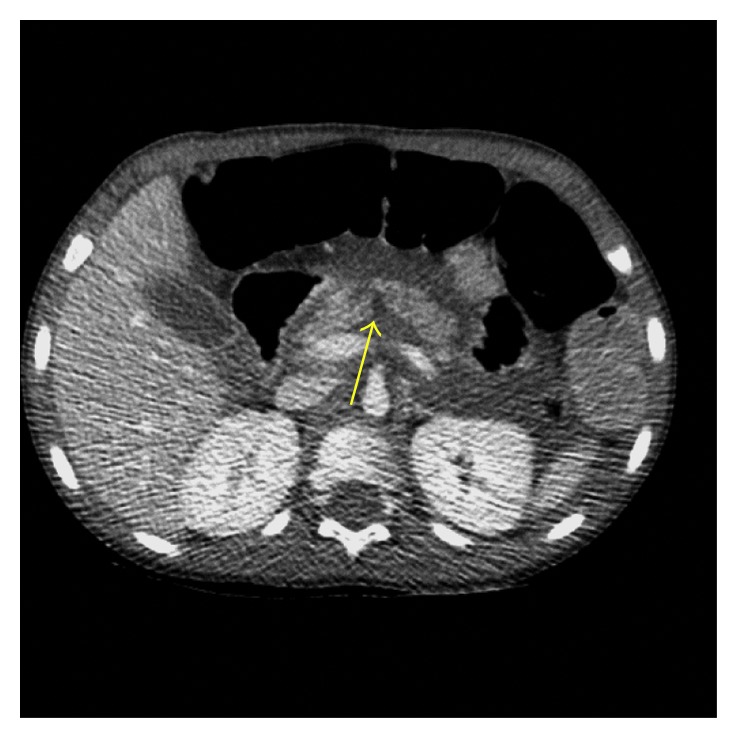
CT of abdomen showing linear low attenuation within the pancreatic body.

**Figure 2:**
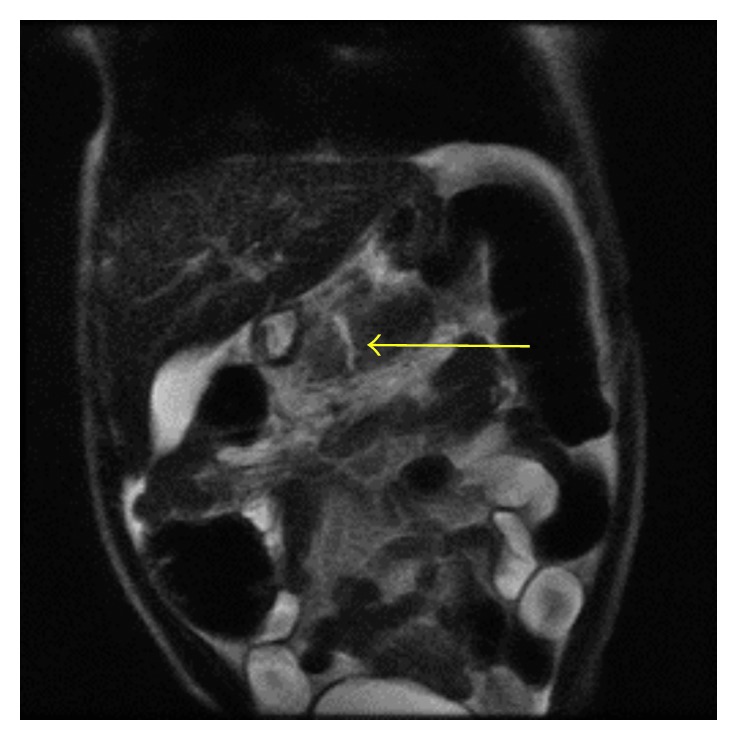
MRI showing complete transection through the proximal portion of the body of the pancreas.

Laboratory results were significant for lipase 9373 U/L (80–360 U/L), hemoglobin 8.1 (10.3–14.9 g/dL), hematocrit 26.2 (32–42.0%), aspartate aminotransferase (AST) 70 (26–55 U/L), and alanine aminotransferase (ALT) 257 (<56 U/L). His total bilirubin at 0.3 (0.0–0.4 mg/dL) and alkaline phosphatase at 199 (256–369 mg/dL) were both normal. His urinalysis was significant for ketones and protein. Ultrasonography of the abdomen showed a normal appendix, a normal gallbladder, no focal liver lesion, no biliary ductal dilation, and no focal splenic lesion. There was a moderate amount of complex intraperitoneal fluid seen and an incomplete visualization of the pancreas. Heterogeneous nodules were seen related to the posterior margin of the body of the pancreas. Abdominal CT (Figure 1) showed linear low attenuation within the pancreatic body with mild to moderate intraperitoneal fluid, indicating a pancreatic laceration. MRI (Figure 2) showed a complete transection through the proximal portion of the body of the pancreas. There was a hematoma along the posterior margin of the pancreas corresponding to the heterogeneous nodules seen on ultrasound.

The patient was admitted to pediatric surgery and underwent a successful distal pancreatectomy with preservation of the spleen. On further inquiry specific to trauma, the mother stated that she recalled the patient had been playing with his brothers upstairs the night before and might have fallen off the bunk bed. Further history by a child advocacy physician on second inquiry was told by the mother that, the evening prior to presentation, she was aware that the child was crying because he claimed he was hurt by his brother. As he had calmed quickly, she did not think anything serious happened as they often “rough house.” In a later interview, the child disclosed that his older brother (described by their mother as a 12-year-old who was approximately 5 1/2 feet and weighing 120 lbs) punched him in his abdomen causing his injury. The child's parents were separated due to intimate partner violence, and this sibling had been recently very stressed. Previously, he had witnessed significant violence towards his mother.

## 2. Discussion

Pediatric pancreatic injuries are rare and account for less than 2% of all abdominal injuries [1]. Most pancreatic traumas are attributed to traffic accidents involving motor vehicles or bicycles in which there is direct, deep impact on the abdomen with the steering wheel or the handle bars. Because the pancreas is a retroperitoneal organ, it is often spared during minor abdominal trauma. The pancreatic body accounts for the majority of blunt pediatric pancreatic trauma at 65% [1]. Although pancreatic trauma is uncommon, it has high morbidity and mortality [2]. This emphasizes the importance of early detection. In children, ultrasonography is sometimes ordered first due to the potential radiation risks of CT. However, CT is likely preferable since ultrasonography is many times not specific, and even signs of pancreatic injury on CT can be subtle and may include bulky pancreas, heterogeneous enhancement, laceration, peripancreatic fluid, signs of pancreatitis, and transection [2].

Major pancreatic injuries are uncommon in children, and the diagnosis often is delayed [3, 4]. However, there are several unique anatomical reasons that make the intra-abdominal organs in children more susceptible to blunt trauma than adults. Children have a less muscular and a thinner abdominal wall. The diaphragm is more horizontal; thus, the liver and spleen are more anterior and less protected by the ribs, which are themselves elastic and very compressible, potentially crushing solid organs below [5]. Abdominal injuries, although rare, are the second commonest cause of fatal physical abuse [6, 7]. In this case, the sibling's blow to the abdomen caused compression of the pancreas between the vertebral column and anterior abdominal wall which resulted in laceration, transection, and hematoma.

Children exposed to intimate partner violence (IPV) are at increased risk of being abused and neglected [8]. They are also more likely to develop adverse health, behavioral, psychological, and social disorders later in life, including violence in their relationships (either as victims or perpetrators) [8]. Sibling abuse is less recognized than spousal or child abuse, undoubtedly influenced by the fact that most studies in sibling abuse rely exclusively on parents' reports [9, 10]. Particularly, siblings who are a great deal larger and/or older than their younger counterparts may in fact be capable of lethal violence towards their victims [11].

Of additional interest, our patient did not have bruising or signs of trauma to the abdomen. Significant visceral injury may present with little or no specific signs, where as few as 12% may have abdominal bruising, emphasizing the need for early consideration as some children may present with nonspecific symptoms, for example, vomiting and irritability [12, 13]. Child abuse should be considered in all cases of nonfatal abdominal injuries presenting to the emergency department. Unless specifically probed, child maltreatment often goes unrecognized, and the victims may be subject to repeated abuse with devastating consequences [14, 15].

## 3. Conclusion

A severe intra-abdominal injury such as pancreatic transection can present with nonspecific symptoms and signs. A low threshold for diagnosing occult trauma is required. Child abuse should be strongly suspected when the clinical history does not match with the severity of injury.
